# Differential impact of hepatitis delta virus replication and expression of viral antigens on the cellular kinome profile

**DOI:** 10.1186/s12964-025-02290-0

**Published:** 2025-06-19

**Authors:** Keerthihan Thiyagarajah, Mirco Glitscher, Kai-Henrik Peiffer, Eberhard Hildt

**Affiliations:** 1 Paul-Ehrlich-Institut, Research Group, D-63325 Langen, Germany; 2 Division Gastroenterology, University Hospital, Frankfurt am Main, Germany; 3 Division Gastroenterology, University Hospital Münster, Münster, Germany; 4 Digital Health Cluster, University of Potsdam, Hasso-Plattner-Institut, Campus III / Rudolf-Breitscheid-Str. 187 / D-14482, Potsdam, Germany

**Keywords:** SHDAg, LHDAg, Host-factors, Pathway analysis, Virus-host interplay, Viral life cycle, Hepatitis D virus

## Abstract

**Background:**

An infection with hepatitis D virus (HDV) is considered the most extreme form of viral hepatitis. Infection with HDV elicits strong increases in inflammation and hepatic injury, therefore pushing liver cirrhosis and development of hepatocellular carcinoma (HCC). Despite this, little is known on how HDV influences the host-cell’s equilibrium. As the pathogenesis is majorly driven by host-responses, a deep understanding is required in terms of how signalling cascades are modulated by the virus in order to identify targets for preventive and therapeutic strategies. Accordingly, this study aims to establish the kinome profile for HDAg-expressing and HDV-replicating cells which could serve as base for future research characterizing HDV-host interaction.

**Methods:**

We performed kinome profiling in Huh7 cells ectopically expressing the two HDV protein isoforms S- and LHDAg or replicating HDV genomes. Significantly deregulated kinases were identified using an array-based screening.

**Results:**

The different HDAg isoforms revealed a differential impact on the overall signalling landscape predominantly in nucleoplasm. Enrichment analyses indicated that HDAg and HDV-replication elicit kinomic changes overlapping with footprints of several diseases such as viral carcinogenesis and HCC. The responsible kinases therefore present promising targets of intervention. Moreover, pathways of innate immunity, inflammation, growth-factor-response yet also distinct modulatory signalling cascades were identified. Most prominently, the MAPK- and PI3K-Akt-cascades were affected by all experimental conditions. Within these cascades AKT1, GSK3A and PRKACA were identified as the most influential hits. A hierarchical pathway map of identified deregulated kinases indicated major changes in inflammatory processes, cell cycle control and metabolic control.

**Conclusions:**

A detailed analysis of the impact of HDV on the cellular kinome was established. Based on this, host-factors, single hits and even entire signalling cascades were identified. These advance understanding of HDV life cycle, and support development of novel therapeutics, yet also help to assess pathogenic processes.

**Supplementary Information:**

The online version contains supplementary material available at 10.1186/s12964-025-02290-0.

## Background

Hepatitis D is a life-threatening disease caused by the hepatitis D virus (HDV). Severe liver damage leading to rapid progression of liver cirrhosis and ultimately to hepatocellular carcinoma (HCC) are its hallmarks. It therefore is considered the most severe form of viral hepatitides [1]. HDV is a 37 nm sized, enveloped RNA virus harboring a small genome of 1.7 kilobases, which does not encode for surface proteins. Thus, HDV is considered as a satellite virus. It relies on a helper virus providing these for envelopment and subsequent spread: the hepatitis B virus (HBV). HDV infections therefore only occur in co-infection with HBV [2–4]. Strikingly, a co-infection doubles the risk of developing liver cirrhosis in comparison to HBV mono-infections and increases chronification up to 80%. Consquently, chronically HDV-infected individuals display a survival rate of only 49% in a years’ timeframe [5–9]. There is only one anti-HDV drug conditionally approved by the European Medicines Agency. Bulevirtide, a myristoylated, synthetic peptide encompassing amino acids 2–48 of the PreS1 domain of the large HBV surface antigen (LHBs), competitively blocks the viral entry receptor Na^+^-taurocholate co-transporting polypeptide (NTCP) of both HBV and HDV [10–12]. Considering severity of HDV-infection, the limited treatment option presents as a devastating health crisis, as clearly indicated by the recent Global Hepatitis Report [13], and is due to a lack of understanding viral life cycle and thus the pathogenic mechanism of HDV. Several studies concluded that HDV-driven pathogenesis is mainly triggered by the host’s immune-response [14]. However, the only protein encoded by the HDV genome is a nucleoprotein termed hepatitis delta antigen (HDAg). It exists in two isoforms being encoded on the same open reading frame separated by a stop codon. Initially, only a 24 kDa small-HDAg (SHDAg) isoform is expressed. Over time, host-mediated RNA editing leads to a stop codon read-through and ultimately to a C-terminal extension of 19 amino acids, resulting in the expression of the large-HDAg protein (LHDAg) [15–20]. Both HDAg isoforms are nuclear histone-like proteins containing RNA-binding domains forming ribonucleoprotein complex (RNP) with viral RNA [21–23]. Both isoforms have distinct and partially opposing functions in the viral replication cycle. SHDAg facilitates HDV genome replication, while LHDAg represses genome replication and instead mediates envelopment and release [15–17, 24]. The HDAg isoforms are subjected to a variety of post-translational modifications including isoprenylation, acetylation, methylation and phosphorylation [25]. Previous studies indicated that the specific protein functions of HDV are tightly regulated by these post-translational modifications, in particular by their phosphorylation status [26–32]. During viral infections, for instance with HBV, these post-translational modifications are affected in favor of the viral replication cycle, impacting the host cell equilibrium and exacerbating viral pathogenesis [33–36]. In line with that, several proteomic and transcriptomic data revealed that the HDAg isoforms majorly dysregulate cellular functions and promote tumorigenesis via their intrinsically disordered nature, thus enabling flexible interaction with a variety of host proteins– many of which giving rise to yet-to-be identified processes driving pathogenesis [37–40]. In this regard, it is very likely that the HDAg proteins also majorly impact the kinome profile of host cells in favor of viral replication and in terms of managing their differential function therein. Considering, that the kinomic landscape is a prominent regulator of cellular processes and has a critical impact on pathogenesis, in this study we focused on investigating the impact of the HDAg isoforms on the kinome of the host cells. In particular, we wanted to decipher intracellular signaling cascades which are altered by sole expression of both HDAg isoforms through ectopic expression and subsequent kinome profiling. Additionally, a cDNA clone of the HDV genome also was used to investigate the impact of the HDV RNP complex and viral genome replication. The experimental design was chosen to study the specific effect on the kinome profile of HDV replication and/or of HDV-specific proteins SHDAg and LHDAg in the absence of HBV, which on the one hand is a limitation yet on the other hand facilitates a cleaner look on the deregulation specifically exerted by HDV alone. We thereby aimed to generate a solid base for understanding the viral life cycle and possible druggable targets with a focus on an early time point of an HDV-infection thus pointing out the beginning of host-deregulation.

## Materials and methods

### Cell culture and transfection

Huh7 cells were maintained in Dulbecco´s Modified Medium (DMEM) supplemented with 10% Fetal calf serum (Bio & Sell, FBS.S0615), 2 mM L-glutamine, 100 µg/ml Streptomycin and 100 units/ml penicillin at 37 °C in a humidified atmosphere containing 5% CO_2_.

Transfection of HDAg isoforms, empty pcDNA3.1(-) vector as negative control as well as the pSVLD3 plasmid, a cDNA clone encoding a trimer of the HDV genome derived from an HDV genotype I infected woodchuck [41] representing our HDV replication condition was performed in 6-well plates. For immunofluorescent stains, cells were seeded on coverslips. Transfection was performed at a cell confluency of 70% wth the transfection reagent FuGENE^®^ HD (Promega) in a 1:3 ratio according to the manufacturer’s instructions. 16 h post-transfection cells were washed once with PBS and media was exchanged with fresh cultivation media and harvested 72 h post-transfection (3 dpt; three days post transfection).

### Plasmid construction

HDAg isoforms were cloned into pcDNA3.1(-) through sequential amplification from the aforementioned pSVLD3 plasmid using the Q5 DNA polymerase (NEB). Restriction enzyme sites and C-terminal StrepTagII alongside a GC-linker were incorporated via primers (Table 1). Amplicons were purified via agarose gel electrophoresis and subsequent gel-extraction. NheI, BamHI and NotI (NEB) in combination with a T4 DNA Ligase (Thermo Scientific) were used to assemble constructs.

**Table 1:** Primer sequences for generation of Recombinant HDAg constructs

**Primer 1**	AAAGCTAGCATGAGCCGGTCCGAGTCGAGGAA
**Primer 2**	CCGCCACCTTTTTCAAATTGGGGGTGTGACCAAGCACTTCCACCTCCCCCGGATCCTGGAAATCCCTGGTTTCCCCTG
**Primer 3**	AAAGCGGCCGCTCATCACTTCTCGAACTGCGGGTGGCTCCACGCGCTGCCACCAGAGCCACCACCACCGGAGCCGCCACCTTTTTCAAATTGG
**Primer 4**	AAAAGCTAGCCACCACCACCACCACCACGAGCTCGCAGCCGCGATGAGCCGGTCCGAGTCGAGG
**Primer 5**	AAAAGGATCCCTGGGGTCGACAACTCTGGGGAGAAAAGGGCGGATCGGCTGGGAAGAGTATATCCCATGGAAATCCCTGGTTTCCCCTG

### SDS-PAGE and western blot analysis

Cells were lysed using radioimmunoprecipitation assay (RIPA) lysis buffer (50 mM Tris, 150 mM NaCl, 0.1% (w/v) SDS, 0.5% (w/v) sodium deoxycholate, 1% (v/v) Triton X-100, pH 7.2) prior to sonication and assessment of protein concentration via a Bradford Assay (Thermo Scientific). 45 µg of total protein were loaded on a 14% SDS- polyacrylamide gel. Proteins were then transferred onto a polyvinylidene fluoride (PVDF; Carl Roth) membrane by conducting a semi-dry western blot method. Membranes were blocked with 5% (w/v) skim-milk powder (Carl Roth) dissolved in TBS-T (TBS supplemented with 0.05% Tween20) for 1 h at room temperature and probed with an HDAg-specific antiserum, which was generated in our lab via immunization of rabbits with recombinantly expressed HDAg (Seramun Diagnostica GmbH; dilution: 1:2,500) or with an anti-StrepTagII antibody (Novus Biological; dilution: 1:1,000) overnight at 4 °C. Membranes were then washed with TBS-T and incubated with IRDye680RD anti-rabbit IgG (LI-COR Biosciences) secondary antibody for 1 h at room temperature (dilution: 1:5,000). Membranes were washed and detected using a LI-COR Odyssey CLx infrared imager (LI-COR Biosciences) and analyzed using the Studio Light Imaging software (v.5.2, LI-COR Biosciences).

### Immunofluorescence microscopy

Cells were fixed by incubation with 4% Para-formaldehyde (Sigma Aldrich) in PBS for 20 min, which was followed by permeabilization for 10 min using 0.5% (v/v) Triton-X-100 (Sigma Aldrich) in PBS. Cells were blocked with 5% BSA (Carl Roth) in PBS-T (PBS supplemented with 0.05% Tween-20) for 1 h and probed with abovementioned HDAg-specific antiserum (dilution: 1:200) for 1 h. After washing with TBS-T, samples were probed with AlexaFluor^®^ 594-conjugated anti-rabbit IgG (Invitrogen; dilution: 1:1000) 250 ng/ml 4′,6-diamidin-2-phenylindole (DAPI; Carl Roth) for 1 h. Cells were again extensively washed and mounted on microscope slides using Mowiol mounting medium. To determine transfection efficiency, stained cells were imaged using the Nikon Ti-U E20L80 microscope (Nikon Metrology GmbH, Alzenau, Germany) and a Mono D2-Qi2 camera. Transfection efficiency was estimated by quantifying total cell count (DAPI) and HDAg positive cell count using Fiji [42]. For subcellular localization analysis, cells were imaged with the Leica SP8 confocal laser scanning microscope (CLSM) (Leica, Wetzlar, Germany) using a 63x magnification oil immersion objective (numerical aperture = 1.4). Images were recorded with an image size of 1024 × 1024 pixels, scan speed of 200 and pinhole dimension of 1.0 airy units. Z-stacks were recorded with a step size of 250 nm. 3D reconstruction was performed with the LASX software (Leica, Wetzlar, Germany). All images were processed by applying the lightning process tool prior to 3D reconstruction. Transfection efficiency was determined by counting nuclei and HDAg-positive signals via Fiji.

### Kinome profiling

Cells were lysed by M-PER Mammalian Protein Extraction Reagent (Thermo Scientific) supplemented with Halt Protease Inhibitor Cocktail (Thermo Scientific) and Halt Phosphatase inhibitor Cocktail (Thermo Scientific) according to manufacturer´s instructions for 15 min on ice. Lysates were cleared twice through centrifugation for 15 min at 16,000 x g and 4 °C and stored at -80 °C. Total protein concentration was determined using the Pierce BCA Protein Assay Kit (Thermo Scientific). Subsequently, kinome profiling was performed as described previously [43] in technical and biological duplicates using the PamStation 12 (PamGene International). In brief, the PamStation^®^12 instrument (PamGene International) was used and operated with the Evolve2 software. The system works with peptide-arrays on a ceramics surface. 13-mer peptides, one type of peptide being concentrated in one spot of the array surface, are derived from known phosphosites of host-proteins being target to a known set of host-kinases present in lysates covering signaling cascades. Peptide arrays were measured for substrate-peptides containing tyrosine phosphosites (PTK) or serine- and/or threonine-containing phosphosites (STK) in separate runs, covering a total of 265 peptides. Substrate phosphorylation is carried out in the presence of reaction mixes supplemented with ATP (PamGene International). Imaging is performed with fluorophore-conjugated antibodies raised against phosphor-tyrosine, -serine or -threonine with the help of a CCD camera. Relative changes in peptide-phosphorylation (spot-intensity) was subsequently calculated with the BioNavigator v6.3.67 software and referred to respective controls (lysates of mock-transfected cells).

### Data processing and statistical analysis

Raw data of peptide phosphorylation was processed via variance stabilizing normalization (VSN) and corrected for batch effects by applying a ComBat model via the BioNavigator v6 software (PamGene International). This was followed by prediction of upstream kinase activity based on the database PhosphoNET as described previously [44], with mock-transfected cells serving as reference. The kinases upstream to these peptides were assessed alongside their activity and statistics. This led to the assessment of a total of 173 host-kinases before applying thresholds. Kinases were considered significantly deregulated above a final score of 1.3. The global kinomic impact then was assessed by calculating the arithmetic mean of the absolute values of change in kinase activity multiplied by the respective final score of all identified, significantly deregulated kinases. Venn diagrams were generated with the help of BioVenn [45]. Enrichment analyses were performed using the g: GOSt tool of gProfiler (version e111_eg58_p18_f463989d, database updated on 25/01/2024) with a threshold of 0.05 on the g: SCS method [46]. Data sources for enrichment included KEGG and Gene Ontology cellular component. Enrichments were performed separately for each experimental condition. In case of GO-terms, overarching terms within the ancestor chart were prioritized for depiction. In case of KEGG pathways, hits were clustered according to them reflecting a clinical syndrome/morbidity, an infection, a cancer type or a classical signaling pathway. Statistics for clusters of kinases were performed using a Shapiro-Wilk test (normality) a Kruskal-Wallis test (significance) via GraphPad Prism 9. Network analyses were performed via NetworkAnalyst v3.0 [47] using the gene list input without an added background. A Steiner-Forest network was built as signaling network (part of Gene Regulatory Networks; GRN) based on SIGNOR 2.0 [48], which was prioritized over a minimal network as for its further condensation, thus little need of background genes not being part of our kinomic hit list. Degree and betweenness of nodes as well as a Fruchterman-Reingold network were extracted, with the first being correlated to one another using GraphPad Prism 9 and the latter being edited for representation using Inkscape. Raw data, enrichment analyses, network analysis and GO-terms/KEGG-entries used in generating the data and figures are available at the following hyperlink to Mendeley Data: https://data.mendeley.com/preview/xz39m5vzm9?a=670fe1ca-4df0-4c54-8ccb-18a0dc9a0095.

## Results

### HDAg isoforms have a differential Kinomic impact

**Fig. 1:**
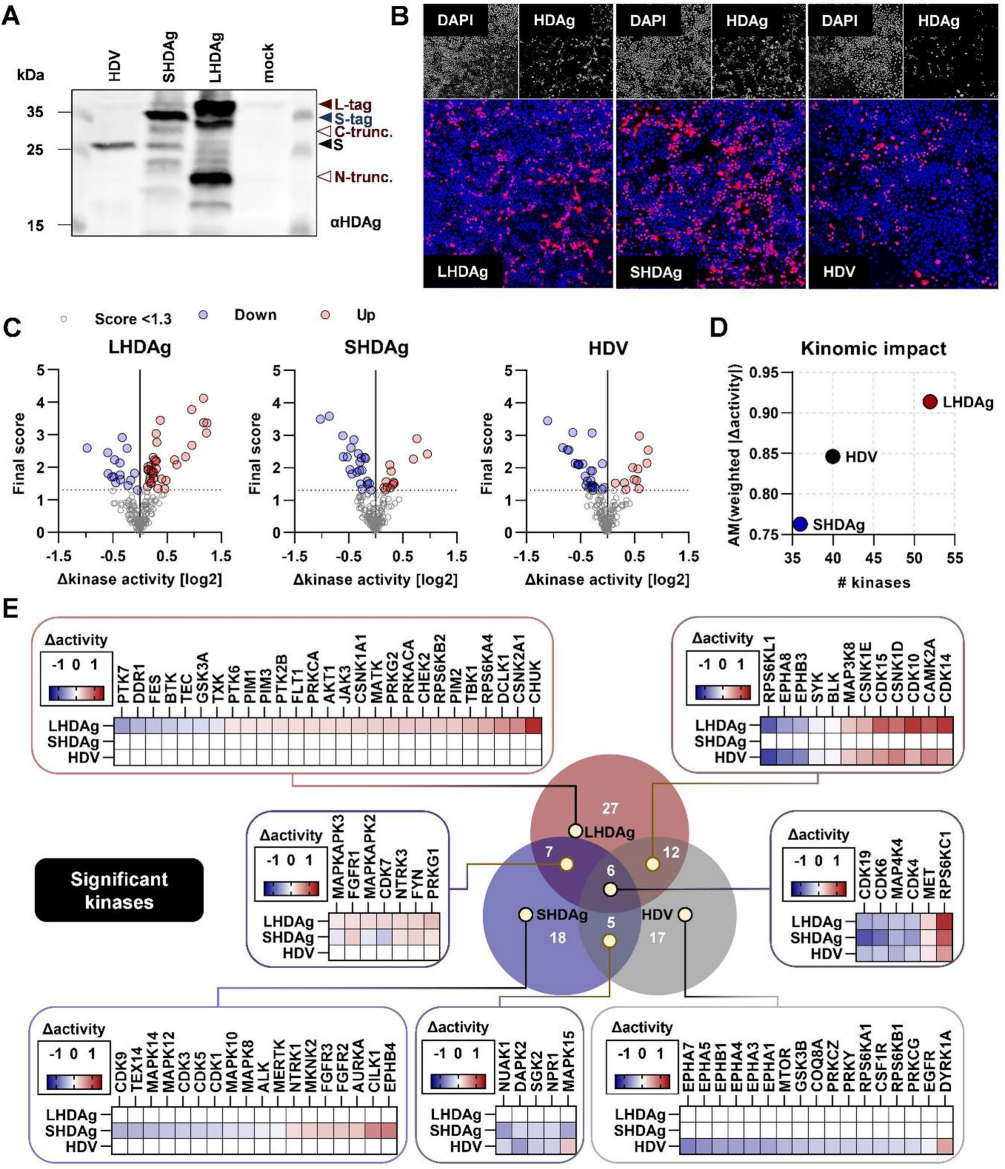
Expression of L-/SHDAg and replication of HDV differentially impacts the kinome of Huh7 cells. (**A**) Representative WB of cell lysates of S/L-HDAg-expressing and HDV-replicating Huh7 cells at 3 dpt; arrows represent different isoforms; L-tag: full-length, tagged LHDAg; C-trunc.: C-terminally truncated LHDAg; N-trunc.: N-terminally truncated LHDAg; S-tag: full-length, tagged SHDAg; S: endogenous SHDAg in HDV replicating cells. (**B**) Representative immunofluorescent stains of Huh7 cells expressing L-/SHDAg or HDV-replication at 3 dpt; blue: DAPI; red: HDAg. (**C**) Volcano plots of kinases identified in kinome screening as referred to mock-transfected cells at 3 dpt; one dot = one kinase; dashed line: threshold (1.3); blue dots: downregulated; red dots: upregulated; gray dots: below threshold. (**D**) Global kinomic impact as assessed by plotting the number of differentially regulated kinases (x-axis) and the arithmetic mean (AM) of weighted, absolute Δkinase activities for significant kinases in (**C**). (**E**) Entirety of identified kinases along with their change in activity; coloring of heat maps represents change in activity in log2 space. Venn diagram generated using BioVenn

To study the impact of HDV viral proteins and intact HDV genome replication on cellular signaling cascades, Huh7 cells were transfected with recombinant variants of either S- or LHDAg or with a three-fold HDV full genome cDNA clone encoded on a vector, termed pSVLD3. Initial Western blot analysis verified successful expression. Interestingly, HDV-replication resulted in sole expression of SHDAg at 3 days post-transfection (dpt), whereas recombinant S- and LHDAg showed additional protein bands, indicating truncations (Fig. 1A) as visible in immunoblots probed for both HDAg and the C-terminal tag (Additional file 1, Fig. S1A). Transfection efficiency was assessed by immunofluorescence microscopy demonstrating at least 30% (Additional file 1, Fig. S1B).

Kinome analyses were performed based on active kinases in cell lysates phosphorylating peptide arrays. Here, Δkinase activity is defined as the relative change in activity as compared to mock-transfected cells, while the final score serves purposes of thresholding (Fig. 1C). Out of a set of 173 host-kinases, 52 (LHDAg), 36 (SHDAg) and 40 (HDV) kinases were identified to be significantly deregulated. Generally, recombinant LHDAg affected the highest number of kinases in line with displaying the highest expression (Fig. 1A). Over 50 kinases were dysregulated, with the greatest impact on the kinomic landscape as of the average absolute weighted change in kinase activity above 0.9. In contrast, SHDAg expression dysregulated only 36 kinases and had the lowest kinomic impact with less than 0.8, although displaying the highest transfection efficiency (Additional file 1, Fig. S1B). In comparison, HDV-replication resulted in dysregulation of 40 kinases with a kinomic impact of 0.85 (Fig. 1D). Only a subset of kinases is similarly deregulated by all three experimental conditions, which include downregulations of CDK4/6/19 and MAP4K4 and upregulations of MET and RPS6KC1 (Fig. 1E). Top deregulated kinases, included strong upregulations of CHUK, CSNK2A1 and DCLK1, whereas PTK7 and DDR1 were strongly downregulated in case of LHDAg. SHDAg strongly upregulated EPHB4 and CILK1 and downregulated CDK9 and TEX14. HDV-replication resulted in strong upregulation of DYRK1A and downregulation of EPHA7 and related isoforms. Importantly, several kinases were dysregulated in opposing directions: MAPK15, MAPKAPK2/3 and CDK7. Interestingly, the general footprint of kinomic impact in HDV-replication was more similar to that of recombinant LHDAg-expression than that of recombinant SHDAg-expression (Fig. 1E) and does not strictly correlate with protein levels or transfection efficiency.

### Nucleoplasmic kinases are dysregulated in opposing directions by SHDAg and LHDAg

**Fig. 2:**
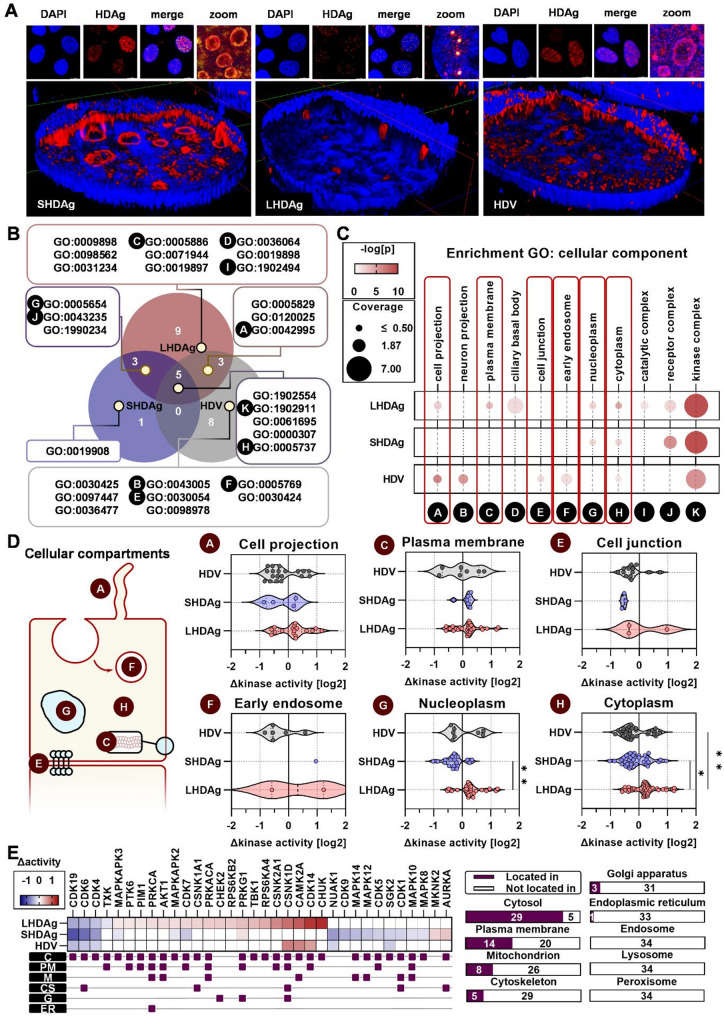
Isoforms of HDAg localize differently in the nucleus leading to a differential impact on nucleoplasmic kinases. (**A**) Representative micrographs and 3D-reconstructions of immunofluorescent stains in Huh7 cells expressing L-/SHDAg or replicating HDV at 3 dpt; blue: DAPI; red: HDAg; zoom: magnified view of merge. (**B**) Enriched GO-terms using Gene Ontology; GO-IDs given; letters indicate selected; Venn diagram generated using BioVenn. (**C**) Enrichment statistics of (B); color: -log(p); size: coverage of GO-term entries by identified kinases in %; rectangles: selected GO-terms for (**D**). (**D**) Global change in kinase activity of marked GO-terms in (**C**); one dot represents one kinase; data tested for normality using Shapiro-Wilk test; *p < 0.05, **p < 0.01, Kruskal-Wallis test. (**E**) Kinase activities of kinases located in the nucleoplasm (GO:0005654) in log2 space along with alternate localizations as of GO-terms (Cytosol, C: GO:0005829; Plasma membrane, PM: GO:0005886; Mitochondrion, M: GO:0005739; Cytoskeleton, CS: GO:0005856; Golgi apparatus, G: GO:0005794; Endoplasmic reticulum, ER: GO:0005783; Endosome: GO:0005768; Lysosome: GO:0005764; Peroxisome: GO:0005777)

The differential kinomic impact between the three experimental settings, may be a result of differential subcellular distributions of HDAg isoforms. To address this hypothesis, we next performed CLSM-based 3D reconstruction. All HDAg isoforms localized in the nucleus, yet SHDAg accumulated in outer rims of nucleoli, whereas LHDAg localized solely in nuclear speckles (Additional file 1, Fig. S1C). Interestingly, cells replicating the HDV-genome showed both distribution patterns (Fig. 2A).

Based on deregulated kinases, enrichment analyses using Gene Ontology (GO)-term cellular component, determining a kinase’s subcellular localization based on curated databases, revealed putative impacts on different organelles. The overarching, most heavily affected organelles were plasma membrane, cell projection, cell junction, early endosome, cytoplasm and importantly nucleoplasm (Fig. 2B-C). Extents of dysregulations of kinases at the plasma membrane, in cell junctions or at cell projections were comparable between experimental settings. In contrast, kinases in the nucleoplasm and the cytoplasm were predominantly upregulated by LHDAg, whereas SHDAg resulted in predominant downregulations. Notably, the global difference in kinase activity of nucleoplasmic kinases was even more significant as compared to cytoplasmic kinases (Fig. 2D). In detail, CDK14, CAMK2A, CSNK1D were strongly upregulated by both LHDAg and HDV-replication. MKNK2, AURKA were moderately upregulated, whereas MAPKAPK2 and CDK7 were moderately downregulated by SHDAg. NUAK1 and CKD9 were downregulated upon both SHDAg-expression and HDV-replication. Moreover, CDK19, CDK6, and CKD4 were strongly downregulated under all three conditions (Fig. 2E). As the identified nucleoplasmic kinases do not solely localize to this compartment, further GO-terms were identified being host to these to assess on how HDAg, as a nuclear stimulus, may affect other organelles in an inside-out signaling scenario. Especially the cytoplasm yet also the plasma membrane were affected. However, distinct kinases also are part of the mitochondrion, the cytoskeleton, the Golgi-apparatus or the endoplasmic reticulum (ER). These include some CDKs and MAPKs as well as PRKCA, PRKACA, PRKG1, CSNK1D, CHEK2, CAMK2A, AURKA and AKT1 (Fig. 2E).

These findings indicate a correlation of the differing intranuclear localization of HDAg isoforms with the differentiated nuclear kinase activity and most likely even with differentiated signaling cascades in other organelles.

### HDAg isoforms induce different pathogenic pathways accelerating disease progression

To investigate the impact on pathogenesis-associated cellular pathways and ultimately on disease progression, we performed a pathway enrichment analysis based on the Kyoto Encyclopedia of Genes and Genomes (KEGG) and clustered enriched pathways with respect to their involvement in different morbidities, infections and cancers.

**Fig. 3:**
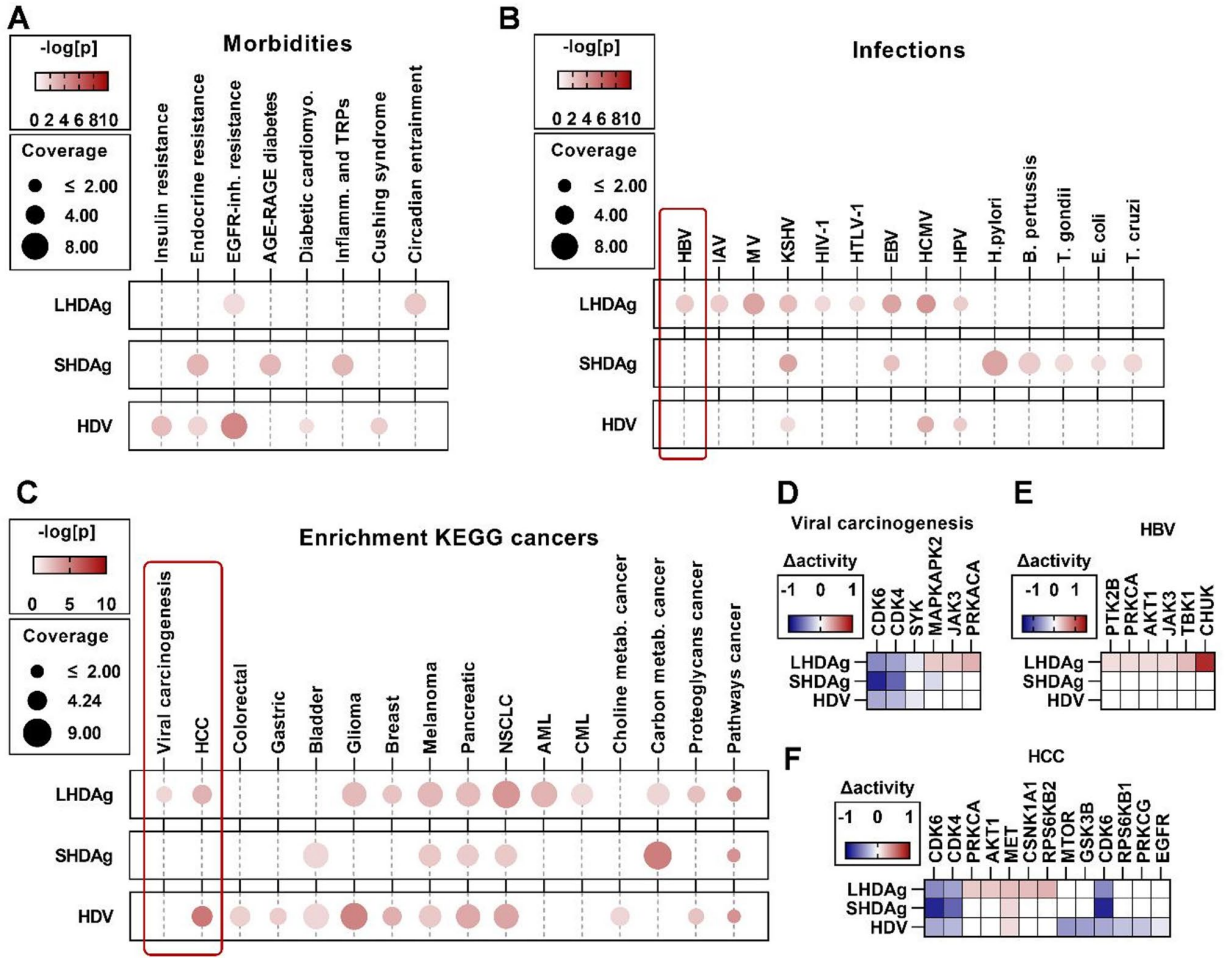
Expression of HDAg isoforms and HDV-replication elicit kinomic footprints similar to various morbidities, infections and cancer types. (**A-C**) Enrichment analysis using KEGG pathways; names of pathways shortened; color: -log(p); size: coverage of KEGG-ID entries by identified kinases in %; rectangles: selected KEGG pathways for (**D-F**). (**D-F**) Change in kinase activities in log2 space; kinases part of enrichments highlighted in red

In terms of pathogenic enrichments, the differential effect of experimental conditions prevailed. Here, overlaps between HDV-replication and HDAg-expression can only be found for endocrine resistance and EGFR-inhibition resistance (Fig. 3A), herpes virus infections (Fig. 3B) and certain types of cancers (Fig. 3C). Most importantly, enrichments in HBV-infection and viral carcinogenesis were identified for LHDAg-expression and a shared enrichment in HCC upon both LHDAg-expression and HDV-replication were identified (Fig. 3B-C). In particular, LHDAg-expression led to strong upregulation of PTK2B, PRKCA, AKT1, JAK3, TBK1 and CHUK, which are also dysregulated during HBV-infection (Fig. 3E). In terms of viral carcinogenesis and HCC, all three tested conditions strongly downregulated CDKs and upregulated MET. Additionally, LHDAg-expression strongly upregulated PRKCA, AKT1, CSNK1A1 and RPS6KB2, whereas HDV-replication downregulated MTOR, GSK3B, RPS6KN1, RPKCG and EGFR (Fig. 3D and F).

In summary, enrichment of pathways linked HDAg-expression or HDV-replication to various pathogenesis and especially to viral carcinogenesis and HCC-development.

### MAPK and PI3K signaling cascades are majorly dysregulated by HDAg-expression and HDV-replication

**Fig. 4:**
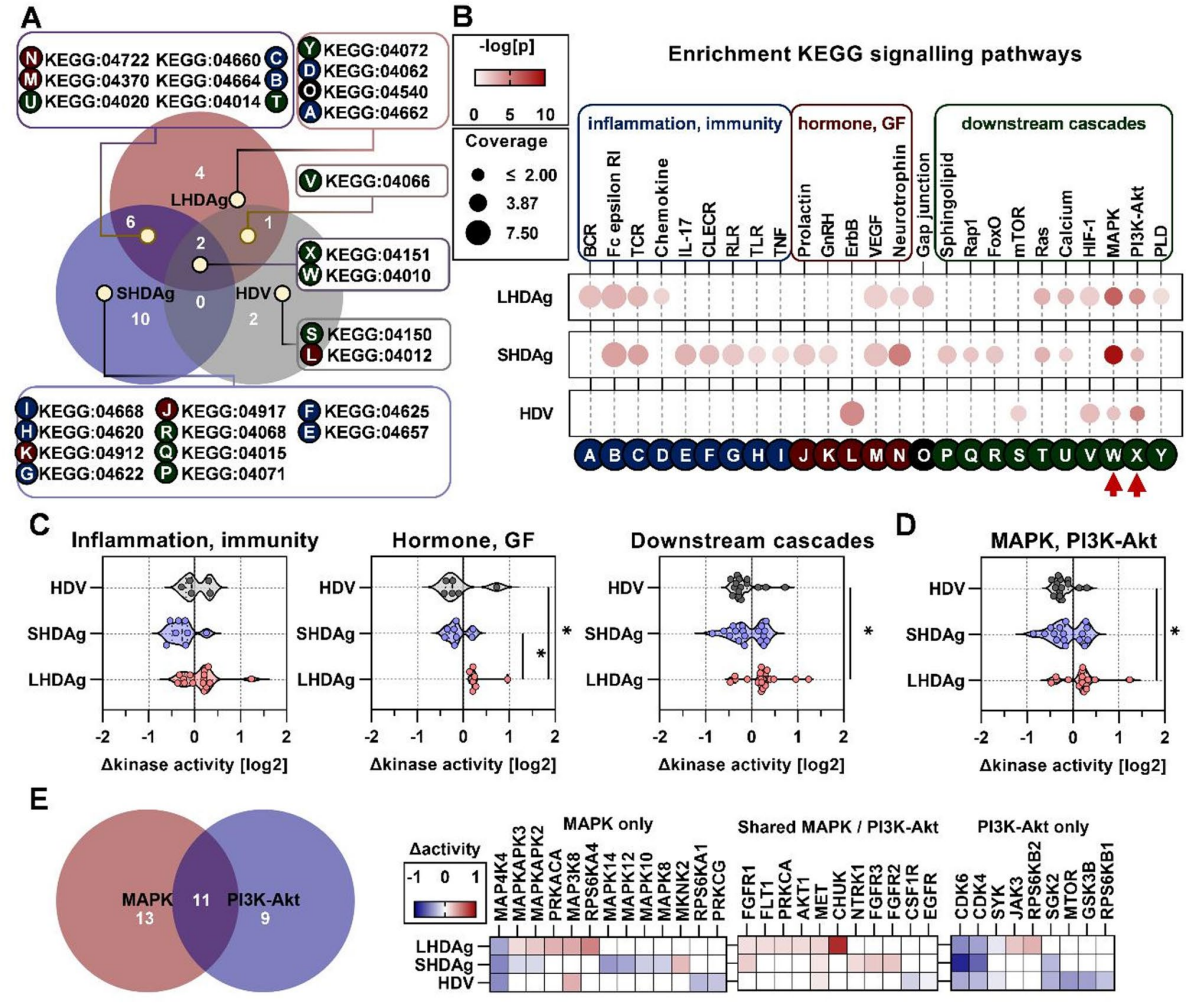
Expression of HDAg isoforms and HDV-replication differentially impact several host-pathways and share an impact on MAPK and PI3K-Akt signaling. (**A**) Enriched signaling pathways using KEGG pathways; KEGG-ID given; letters indicate pathway in (**B**). (**B**) Enrichment statistics of (**A**); color: -log(p); size: coverage of KEGG-ID entries by identified kinases in %; arrows: enriched in all experimental settings; rectangles and coloring: pathway clusters. (**C-D**) Global change in kinase activity of clusters in (**B**); one dot represents one kinase; data tested for normality using Shapiro-Wilk test; *p < 0.05, Kruskal-Wallis test. (**E**) Identified kinases in MAPK- (hsa04010) and/or PI3K-Akt- (hsa04151) signaling along with change in activity; coloring of heat maps represents change in activity in log2 space. Venn diagrams generated using BioVenn

To elucidate underlying regulatory mechanisms relevant for HDV-associated pathogenesis, we assessed the affected individual signaling cascades within the host-cell. We clustered enriched KEGG-pathways into inflammation- and immunity-associated or hormone and growth factor-associated pathways and their downstream cascades fine-tuning responses to external and internal stimuli (Fig. 4).

SHDAg-expression showed the greatest impact on signaling pathways throughout all clusters, whereas HDV-replication showed the least (Fig. 4B). Comparison of changes in global kinase activities over these different clusters revealed that, just as in case of cellular components, LHDAg-expression elicited significantly different changes as compared to SHDAg-expression and HDV-replication (Fig. 4C). This also held true for MAPK- and PI3k-Akt-signaling, which presented as the only enriched pathway shared by all experimental settings (Fig. 4B). Kinases activities within these pathways were significantly higher upon LHDAg-expression as compared to HDV-replication, with SHDAg-expression presenting an intermediate activity profile (Fig. 4D). Comparison of kinases exclusive for either signaling pathway or exclusive to each yet again revealed the differential nature between experimental settings. Generally, kinases of the PI3K-Akt-signaling were predominantly downregulated, shared kinases were upregulated and kinases of the MAPK-signaling displayed a mixed dysregulation (Fig. 4E). Upon closer inspection, consistencies and inconsistencies can be extracted. For example, MAP4K4 and CDK4/6 were downregulated in all experimental settings, while MAPKAPK2/3 were affected opposingly by HDAg isoforms. Apart from these, other MAPKs are predominantly inhibited by SHDAg-expression, while the majority of receptor-kinases are upregulated by HDAg isoforms. Lastly, a diverging dysregulation of ribosomal protein S6 kinase isoforms was observed in case of LHDAg-expression and HDV-replication.

**Fig. 5:**
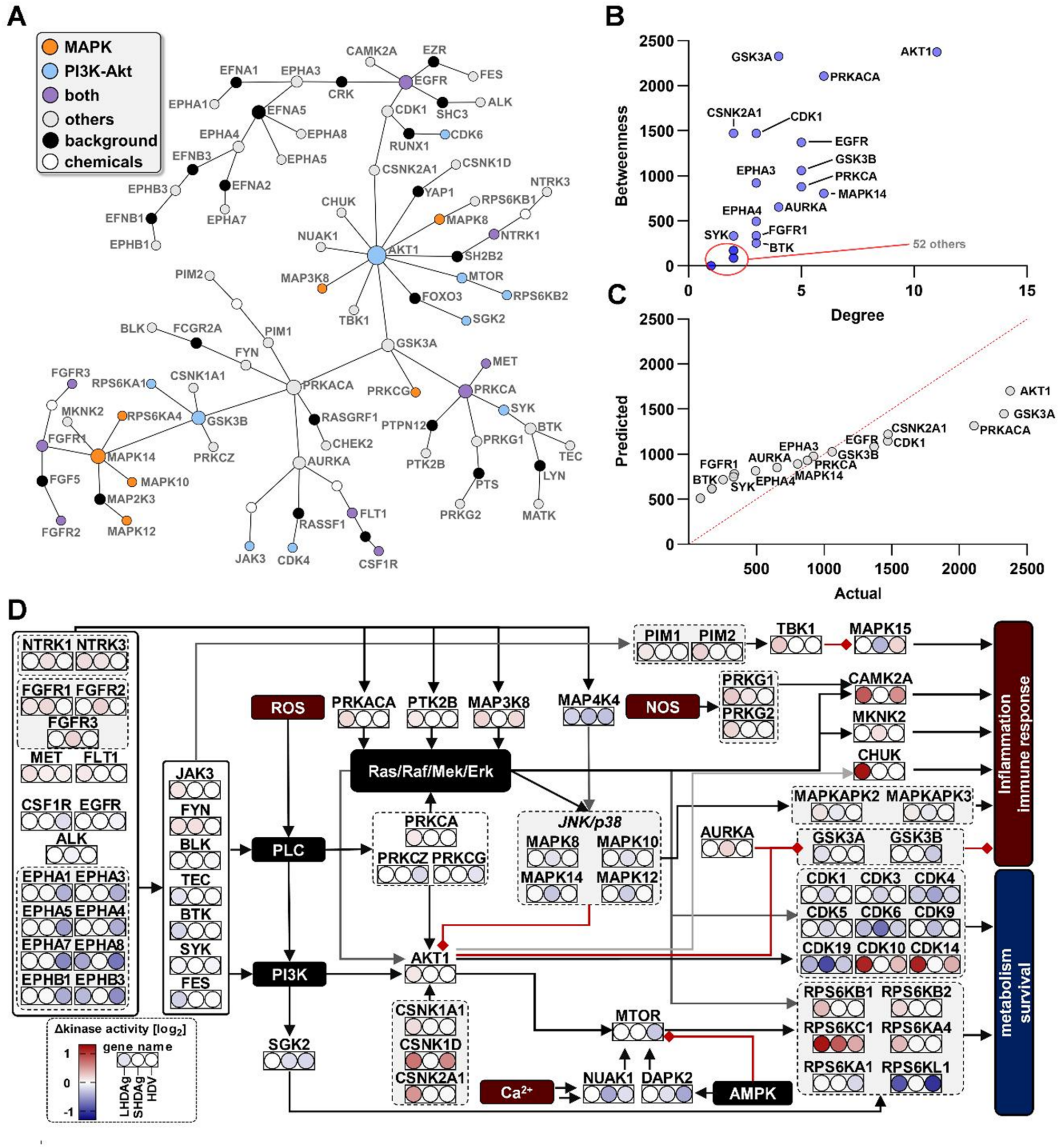
Network analysis and merged pathway map reveal major key-players in kinomic changes elicited by HDAg expression or HDV-replication. (**A**) Gene-regulatory network generated via NetworkAnalyst v3 (SIGNOR 2.0); orange: MAPK-signaling (KEGG: hsa04010); blue: PI3K-Akt-signaling (hsa04151); purple: MAPK- and PI3K-Akt-signaling; grey: other pathways; black: background proteins; white: background chemicals. (**B**) Volcano-like plot of Degree and Betweenness in (**A**); 52 kinases with little/no betweenness/degree. (**C**) QQ-plot of x/y-correlation analysis of (**A**). (**D**) Pathway map of merged cascades in Fig. 4; kinase given along change in activity; black boxes: central connecting nodes not covered by our data; red boxes: internal stimuli; black arrows: activation; red arrows: inhibition

In essence, identification of MAPK and PI3K-Akt signaling as major drivers of the changes in host-signaling landscape seems apparent. To integrate consistencies and inconsistencies observed in Fig. 4 into a signaling map, an undirected network analysis was performed on the basis of the entirety of identified kinases. A focus was set on kinase degree (number of connections of one kinase) and betweenness (how often a kinase acts as bridge to other kinases via the shortest path). Finally, a hierarchical, directed pathway map along with the associated change in kinase activity for each experimental condition was generated (Fig. 5).

In the resulting Steiner-Forest network, members of either MAPK or PI3K-Akt signaling are scattered into all modules apart from the Ephrin-receptors (Fig. 5A). The network further revealed that especially AKT1, PRKACA and MAPK14 present as the most prominent hub-kinases as for their degree being the highest of the 67 included kinases (Fig. 5A-B). Apart from these, EGFR, GSK3B and PRKCA also present an elevated degree as compared to other kinases (Fig. 5B). In terms of betweenness, AKT1, PRKACA and GSK3A present the most prominent hits (Fig. 5B), which also fall out of the range of normal distribution of a degree-betweenness correlation (Fig. 5C), thus highlighting their importance. These gain further relevance in the pathway map (Fig. 5D), where Ras-Raf-Mek-Erk, MAPK and PI3K-Akt-pathways are displayed from top to bottom alongside internal and external stimuli. While the Ras-Raf-Mek-Erk axis is upregulated, the MAPK-related connections are generally downregulated. Interestingly, the classical PI3K-Akt cascade is also upregulated up until the point of AKT1, yet does not lead to a positive continuation onto MTOR. In terms of effectors, the most consistent train of upregulations was found to upregulate innate immunity and inflammation. Here, CAMK2A, MKNK2 and CHUK receive signals from all major axes. However, the effector MAPK15 ultimately is differentially regulated in the experimental conditions and only upregulated in case of an HDV-replication. Further, GSK3 isoforms, inhibiting inflammatory processes, receive an inhibitory signal from both AURKA and the PI3K-Akt axis. Interestingly, HDAg isoforms inhibit the classical JNK/p38 module, which consequently fails to activate MAPKAPK2/3 in case of SHDAg. In terms of metabolic control via RPS6 isoforms and cell-cycle control via CDKs, a mixed picture can be observed. The majority of identified CDKs was inhibited, despite of activating signals. Only CDK10 and CDK14 correlated with an increased activity. In terms of RPS6 isoforms, only RPS6KA1 and RPS6KL1 are fittingly downregulated. As far as the other isoforms are concerned, a strong upregulation could be observed especially for RPS6KC1. These therefore match with signals being passed down from the Ras-Raf-Mek-Erk.

While major regulatory key-kinases could be identified as e.g. AKT, PRKACA and GSK3A, certain points of non-matching signal continuation within the pathway map points out further regulatory mechanisms likely being in place.

## Discussion

Amongst all viral hepatitides, a chronic HBV/HDV co-infection induces the most severe clinical course as marked by an accelerated cirrhosis and progression towards HCC [5–9]. However, only little is known about the intracellular pathogenic mechanisms underlying the severity of an HDV-infection. Therefore, here we provide - to our knowledge - the first thorough analysis of signaling pathway dysregulation in response to the expression of both viral proteins alongside comparing these to an HDV-replication. The type of experimental setup allows for an isolated view on what is caused by HDV itself. Through enrichment and network analyses, we aimed to build a scheme over which each viral component, SHDAg, LHDAg and intact genome replication, modulates the host thus leading to pathogenesis. The cross-talk between HDV and HBV under clinical settings is highly dynamic and thus difficult to entirely reproduce in vitro [49]. With our selective approach, we aimed to investigate pathogenic mechanisms of HDV alone without interference by HBV. We therefore used a well-characterized system for both viruses, Huh7 cells. These also allow picking up changes in intracellular signaling pathways reflected by the kinome profile, although some impairments in innate immunity are in place in these cells. Validating hits in other cell lines may prove useful in the future. Also, we aimed to characterize an early time point as for some of the deregulated mechanisms being very short-lived and easy to miss at later stages [50], which may be addressed by also analyzing top hits over time. This provides the basis to attribute certain pathogenic mechanisms more precisely either to HDV or HBV in clinical settings. As mentioned in the introductory section, several studies characterized the pathogenic traits of HBV monoinfection on the host. In future studies, our results therefore need to be considered before this background in order to deduce pathogenic mechanisms enhancing progression to HCC by HDV-specific deregulation of tumor formation promoting signaling cascades [51].

We identified over 90 significantly dysregulated kinases located in different cellular compartments and being part of various signaling cascades. There is a differential impact exerted by SHDAg, LHDAg and HDV-replication. In general, the HDV-replication exerted only an intermediate impact on the cellular kinome profile compared to SHDAg and LHDAg and the latter two even exerted partially opposing deregulation. In essence, this comes down to the dynamic intraviral network leading to a differentiated kinomic footprint between the selective overexpression of the HDAg protein variants and the HDV replication. This pattern, selected viral components behaving differently as compared to viral replication, is also observable in other viruses such as HBV [52, 53] and is not limited to the kinome but also affects e.g. the host-proteome upon viral infections such as SARS-CoV-2 [54]. Interestingly, this also held true for the landscape of dysregulated nucleoplasmic and cytoplasmic kinases. Upon LHDAg expression, most nucleoplasmic kinases were upregulated, which was opposed by SHDAg.

Previous studies revealed that LHDAg is phosphorylated to a 10-times higher extent as compared to SHDAg, although LHDAg contains only two additional phosphorylation sites [28]. This could be due to the presence of PxxP-motif in the LHDAg-specific extension which could serve as an adaptor of SH-domain harboring kinases such as SFKs. Considering, a recent study showing the broad antiviral effect of specific SFK inhibition on various RNA viruses [55], future studies need to be conducted to assess the antiviral potency of SFK inhibition in HDV infection or through performing studies on PxxP- or phosphorylation site-defective mutant HDAg. It is postulated, that the phosphorylation status might be causative for the partially opposing biological functions of the isoforms [26–28, 31, 56]. The observed hyperphosphorylation of LHDAg might thus explain why it has a more pronounced effect as compared to the other experimental conditions. Obviously, the subcellular localization of kinases gains importance once the subcellular localization of HDAg isoforms is regarded. Both HDAg isoforms are expressed in the cytoplasm and translocated into the nucleus via their nuclear localization signal. It therefore seems straight-forward that their main kinomic impact is concentrated in these two compartments, which we can observe in our datasets. Moreover, even within the nucleus S- and LHDAg show very different localization, namely nucleolus (SHDAg) and non-nuclear speckles (LHDAg). The latter additional harbors a nuclear export signal and thus can be shuttled between nucleus and cytoplasm [57]. SHDAg on the other hand has only a nuclear localization signal [58]. Considering, that most of the nucleoplasmic kinases can also be found in the cytosol, it is unclear if these kinases are impacted in the nucleus or cytoplasm. However, we can postulate that kinases which are impacted in all three experimental conditions such as CDK4/6/19 are impacted in the nucleus, while kinases which are only activated by LHDAg or HDV genome replication such as most of the MAPKs can also be directly impacted in the cytoplasm. Moreover, LHDAg is also prenylated and as a result is anchored on the cytoplasmic face into the ER membrane [59]. Hence, certain kinases such as PRKCA might also be activated directly by LHDAg at the ER. This of course could also be impacted by HDAg degradation thus lacking N- or C-terminal protein motifs for translocation as observed in our immunoblot and in clinics [60, 61]. However, considering that the most prominent localization of LHDAg resided within the nucleus as reflected by our immunofluorescence analysis, we assume that the nuclear LHDAg similar to nuclear SHDAg elicits the highest impact on the kinome profile. In this regard, cytoplasmic kinases can be affected by two means. Either, indirectly by a deregulated signaling cascade originating from the nucleus, or by direct interaction of respective kinases with the HDAg proteins in the nucleus and subsequent shuttling into the cytoplasm. Colocalization studies of kinases with the HDAg proteins dissected by subcellular and subnuclear localization and dynamic analyses, as well as intentional translocation of the HDAg proteins to different compartments could be insightful to understand the relevance of the subcellular distribution of the proteins for deregulation of signaling cascade. Lastly, comparison of alternate localizations of nucleoplasmic kinases gives hints on how a signal originating in the nucleus can still be passed on to other organelles such as mitochondria, which gain relevance as for their involvement in build-up of reactive oxygen species (ROS) and the related pathogenic outcomes [62]. Here, major key-players in a variety of signaling pathways are dysregulated in our study, which include MAPK10/12/14, PRKCA, AKT1, PRKACA, CAMK2A and CDK1. This would leave the conventional point of view we have on a signaling cascade, as it would revert the direction from receptor to nucleus. In fact, the nucleus itself is known to communicate with a variety of organelles including mitochondria, lysosomes, the endoplasmic reticulum, and the Golgi apparatus [63]. This reflects a likely reality and the direction where current views on classical pathway analyses are headed.

To understand the pathogenic mechanism of HDV in more detail, we next analyzed individual signaling cascades via enrichment. Here, inflammatory, immunity-related or hormone- and growth-factor-driven signaling cascades were identified, alongside their downstream effector cascades fine-tuning responses. Of all enriched signaling pathways, only two cascades were enriched in all three conditions, namely the MAPK and the PI3K-Akt pathway. This reflects a central role of these pathways in HDV-replication and host-immune response. Similar to previous observations, LHDAg upregulated most of the kinases within these cascades whereas SHDAg and expression of the HDV genome downregulated these cascades. Considering that SHDAg is involved in HDV-replication, whereas LHDAg inhibits replication and promotes progeny virus assembly and release [15–17], we assume that the shift from replication towards virion assembly and release could be regulated by the PI3K-Akt and MAPK pathway. We observed a strong upregulation of CHUK (IKK-α) in LHDAg expressing cells, which can phosphorylate IκBα and as such mark IκBα for degradation and thereby activate the NF-κB pathway. CHUK on the other hand can be activated through the PI3K-Akt signaling cascade, which only is activated upon LHDAg expression [64]. Studies by Williams et al. further revealed that ectopic LHDAg expression activates NADPH oxidase 4 expression leading to ROS build-up [65]. Elevated ROS levels are also known to activate AKT1 via the PI3K-Akt pathway [66] and are a major driver of genetic instability, thus being of importance when it comes to pathogenic processes. Similarly, this prompts an NF-kB activation by LHDAg, which could be the mediator of increased inflammation. An interesting observation in this context is that CSNK2A1 can also activate AKT1 and its downstream cascades such as NF-κB [67]. Our observation of CSNK2A upregulation by LHDAg might therefore reflect a key factor in HDV replication. In accordance, CSNK2A is seemingly overexpressed in many cancer cell lines such as breast, prostate and colon cancer [67–69]. Moreover, if activation of the NF-κB pathway is an essential step in HDV replication or a result of host-immune response needs to be elucidated further. Nevertheless, constitutive NF-κB activation is strongly associated with HCC progression [70] and may be linked to AKT1-activations observed by us. Noteworthy, several AKT-inhibitors are currently tested as cancer inhibitors. Amongst them Ipatasertib is currently in phase III trials to treat breast cancer and might also be applicable in HDV associated HCC [71].

As mentioned above, the second signaling cascade of interest is the MAPK signaling. Similarly, it was already described to be involved in the HDV replication cycle. For instance, the SHDAg protein has a conserved motif acting as MAPK-substrate, which is phosphorylated by ERK1/2 [27]. ERK1/2 is activated during the MAPK-signaling as well as being an ending point to the Ras-Raf-Mek cascade. The latter also was found to be integrated in our postulated pathway map, as it receives signals from various kinases being deregulated and passes on signals to both PI3K-Akt and MAPK cascades. In particular, both PRKCA and PRKACA seem to be of interest herein. These may promote SHDAg-phosphorylation through ERK1/2, which is crucial for interaction of SHDAg with RNAPII leading to a shift from antigenomic to genomic HDV replication [27, 29]. Overall, these findings line up with our observation of MAPK-signaling being activated upon LHDAg expression. Apart from this, the MAPK-signaling may explain inconsistencies seen for the PI3K-Akt pathway and its downstream effectors. Specifically, we did observe a strong activation of RPS6 isoforms predominantly in LHDAg-expressing cells. Since mTOR was not upregulated in any of the three experimental settings, it is likely that the RPS6 isoforms were activated by the MAPK signaling cascade [72]. RPS6 are generally involved in cell metabolism such as glucose homeostasis and protein translation and as such might promote viral replication, yet al.so cell growth in the context of tumorigenesis [73]. One overlapping example in this context is RPS6KB1. This isoform is upregulated upon LHDAg-expression and similarly is highly expressed in HCC patients. Moreover, expression levels were strongly correlated with tumor size and poor survival prognosis [74]. These examples may present as important link as to why HDV acts so harshly on disease progression.

In essence, many kinases within the PI3K-Akt or the MAPK signaling cascade display pathogenic and oncogenic traits upon constitutive upregulation. However, not all kinases are central and essential in the HDV replication cycle and therefore are ineffective as targets. For this reason, we determined the degree and betweenness of all dysregulated kinases within the two major signaling cascade via a network analysis. Here, we identified 6 top hits, which reflect potential drug targets, namely AKT1, PRKCA, PRKACA, EGFR, MAPK14, and GSK3B. In this regard, Buchmann et al. identified 8 kinase inhibitors, which displayed dose-dependent anti-viral activity against HDV. Amongst the top hits were HA1077 a PRKACA inhibitor, PD 174,265 an EGFR inhibitor, GSKB3 inhibitor which can inhibit both GSK3B and PRKCA [75]. This provides a basis for parts of our analyses, yet al.so points out that the remaining targets require further attention. This also includes kinases not being part of the abovementioned top six hits. In the study performed by Buchmann et al., a Cdk/Crk-inhibitor which inhibits CDK4 and CDK6 also showed anti-HDV activity. Contrary to their screening, in our kinome profile analysis these CDK isoforms were downregulated. Noteworthy, these were part of only 6 kinases which were similarly deregulated in all three experimental settings of our datasets. Interestingly, these kinases are also strongly associated with viral carcinogenesis and HCC, and as such most likely play a central role in HDV pathogenesis. Downregulation of these kinases, will lead to cell cycle arrest [76], which seems to be a hallmark of HDV-infections [77–79]. This HDV-induced cell cycle arrest might be explained by downregulation of CDK4/6 as observed in our analysis. Indeed, a signaling pathway enrichment analysis of dysregulated genes in HDV-HCC specimens compared to HBV-HCC specimens performed by Diaz et al. revealed that cell cycle regulation signaling as well as G2-M DNA damage check-point regulation were exclusively upregulated in HDV-HCC specimens, but not in HBV-HCC specimens [80]. In this regard, we can hypothesize that constitutive CDK4/6 inhibition might lead to mutations enabling escape from cell-cycle arrest and apoptosis and ultimately lead to HCC progression. The discrepancy in our study and that of Buchmann et al. might be explained by certain unspecificity of the inhibitor, compensatory effects by the host or due to different experimental settings in both studies. Nevertheless, the sole identification of CDK4/6 inhibitors still indicates a central role of these kinases in the viral replication cycle. Despite some discrepancies, the screening analysis of Buchmann et al. still is in accordance with our observation, thus emphasizing the power of kinome profile analysis in understanding pathogenic mechanisms and identifying potential drug targets for translational experiments. A first set of promising targets was identified with pathway enrichments resulting in shared kinases being deregulated by both HDV and HBV, namely PTK2B, PRKCA, AKT1, JAK3, TBK1 and CHUK. This highlights that there is at least some overlap with previously described, HBV- specific findings made in interactome analysis conducted by Kar et al. i.e. an activation of the NF-κB pathway mediated by HBV HBx [51]. These overlaying approaches will therefore be extremely useful pinpointing drivers of pathogenesis in future side-by-side experiments.

Aim of the study was to provide a detailed kinome profile of HDAg isoforms expressing cells and HDV genome replicating cells which could serve as base for a deeper understanding of the virus host interaction and for the rational design of antiviral strategies affecting the functionality of defined kinases.

Analysis of HDAg-expression in the absence of HBV co-expression is not unprecedented (for a review see: [81]). We are aware that the focus on HDAg-expression in the absence of HBV coinfection can be considered as a limitation of the study. However, we deliberately chose this experimental setting to ensure the detection of HDV-specific effects in the absence of HBV-specific effects. On the first glance, this might appear as an non-physiologic situation, however it is clear for several reasons that the effect of HBV/HDV coinfection is more than just an additive or cooperative effect of both viruses on the host kinome. There is a very complex interaction between both virus affecting the expression level of the virus, and thereby the kinome profile, making it extremely difficult to dissect effects specific for each virus. Moreover, the crosstalk between the kinomic activities triggered by both viruses affects their replication levels over the time. One example of this is the HDV-dependent activation of Janus kinases that favor on the one hand HDV replication [82] and on the other hand impairs HBV replication. This would lead to a permanently changing kinome activity– the profile would represent a snapshot depending on a lot of partly unknown parameters.

Apart from this, there are technical reasons arguing against a kinome analysis based on coinfected cells. There will be a mixture of either HBV-positive cells, HDV positive and HBV/HDV-positive cells. In light of this it might be difficult to correlate the respective effect with a specific population as the assay is based on total lysate. In light of this the present study abstains to include detailed analyses about the impact of deregulation of selected kinases on viral life cycle and virus associated pathogenesis. This will be addressed in future studies focused on potential targets identified in this study here by kinome profiling. Moreover, this study provides a snapshot of the early stages of HDV infection in one specific hepatic cell line. Considering, that the kinome profile might change during the course of the viral infection and differ between various hepatic cell lines, later time points and other hepatic cell lines need to be investigated in future studies. Further, it needs to be considered that the protein levels although using identical expression vector backbone differ i.e. due to differences in protein degradation rates and/or translation efficiency. These differences were taken in account in our study, however an absolute normalization would have bored the risk of distorting the results. We did not observe a clear correlation between the protein amount and the impact on the kinomic landscape. Thus, we conclude, that the individual HDAg protein variants might have different regulatory capacities on the kinome landscape, which does not necessarily correlate to the respective protein level. Conclusively, in our study we only focused on qualitative trends in the overall deregulation without quantitatively comparing the kinase activity of specific kinases between various conditions.

## Conclusions

The data and analyses set forth in this study present a strong basis for future research. We were able to deduce a variety of host-kinases being deregulated by selective expression of the two HDAg-isoforms or HDV-replication. We identified a variety of interesting targets, which carry the potential to promote both HDV-replication and progression towards HCC. These hits were successfully embedded into greater context, which greatly helps in understanding the underlying mechanisms. Not only will these findings aid basic research, yet also will aid in clinical development of current and future preventive and therapeutic strategies.

## Electronic supplementary material

Below is the link to the electronic supplementary material.

Supplementary Material 1

## Data Availability

Raw data, enrichment analyses, network analysis and GO-terms/KEGG-entries used in generating the data and figures are available at the following hyperlink to Mendeley Data: (https://data.mendeley.com/preview/xz39m5vzm9?a=670fe1ca-4df0-4c54-8ccb-18a0dc9a0095. These will be published upon acceptance and the link will be updated. All other study data are available from the corresponding author on reasonable request.

## Abbreviations

HDV: Hepatitis D virus
HCC: Hepatocellular carcinoma
SHDAg: Small hepatitis delta antigen
LHDAg: Large hepatitis delta antigen
HCC: Hepatocellular carcinoma
HBV: Hepatitis B virus
NTCP: Na^+^-taurocholate co-transporting polypeptide
LHBs: Large HBV surface antigen
RNP: Ribonucleoprotein complex
DMEM: Dulbecco´s Modified Medium
pt: Post-transfection
DAPI: 4′,6-Diamidin-2-phenylindole
CLSM: Laser scanning microsope
VSN: Variance stabilizing normalization
GRN: Gene regulatory networks
GO: Gene ontology
ER: Endoplasmic reticulum
KEGG: Kyoto encyclopedia of genes and genomes
EGFR: Epidermal growth factor receptor
ROS: Reactive oxygen species
