# Supplementary material for: Differential impact of hepatitis delta virus replication and expression of viral antigens on the cellular kinome profile
Source: Cell Commun Signal. 2025 Jun 19;23:294. doi: 10.1186/s12964-025-02290-0 (PMC12180150; doi:10.1186/s12964-025-02290-0)
Supplement: Supplementary file 1 — Supplementary Material 1 [file 12964_2025_2290_MOESM1_ESM.docx]

# Ref: Submission ID 4fa4ec77-d773-4484-a2db-66b3bdf84811 – Additional File 1

**Title**

Differential impact of Hepatitis Delta Virus replication and expression of viral antigens on the cellular kinome profile

**Authors**

Keerthihan Thiyagarajah^a,b,‡^, Mirco Glitscher^a,‡^, Kai-Henrik Peiffer^c^, Eberhard Hildt^a, d,*^

**Affiliations**

^‡^Authors contributed equally

^a^Paul-Ehrlich-Institut, Research Group, D-63325 Langen, Germany

^b^University Hospital, Division Gastroenterology Frankfurt am Main Germany

^c^University Hospital Münster, Division Gastroenterology, Münster Germany

^d^University of Potsdam, Hasso-Plattner-Institut, Digital Health Cluster, Potsdam, Germany

^*^Correspondence

**Correspondence**

Prof. Dr. Eberhard Hildt, email: [eberhard.hildt@guest.hpi.de](mailto:eberhard.hildt@guest.hpi.de), phone+ 49 331 5509-164

Hasso-Plattner-Insttute Digital Health Cluster /Campus III / Rudolf-Breitscheid-Str. 187 / D-14482 Potsdam

# Additional File 1


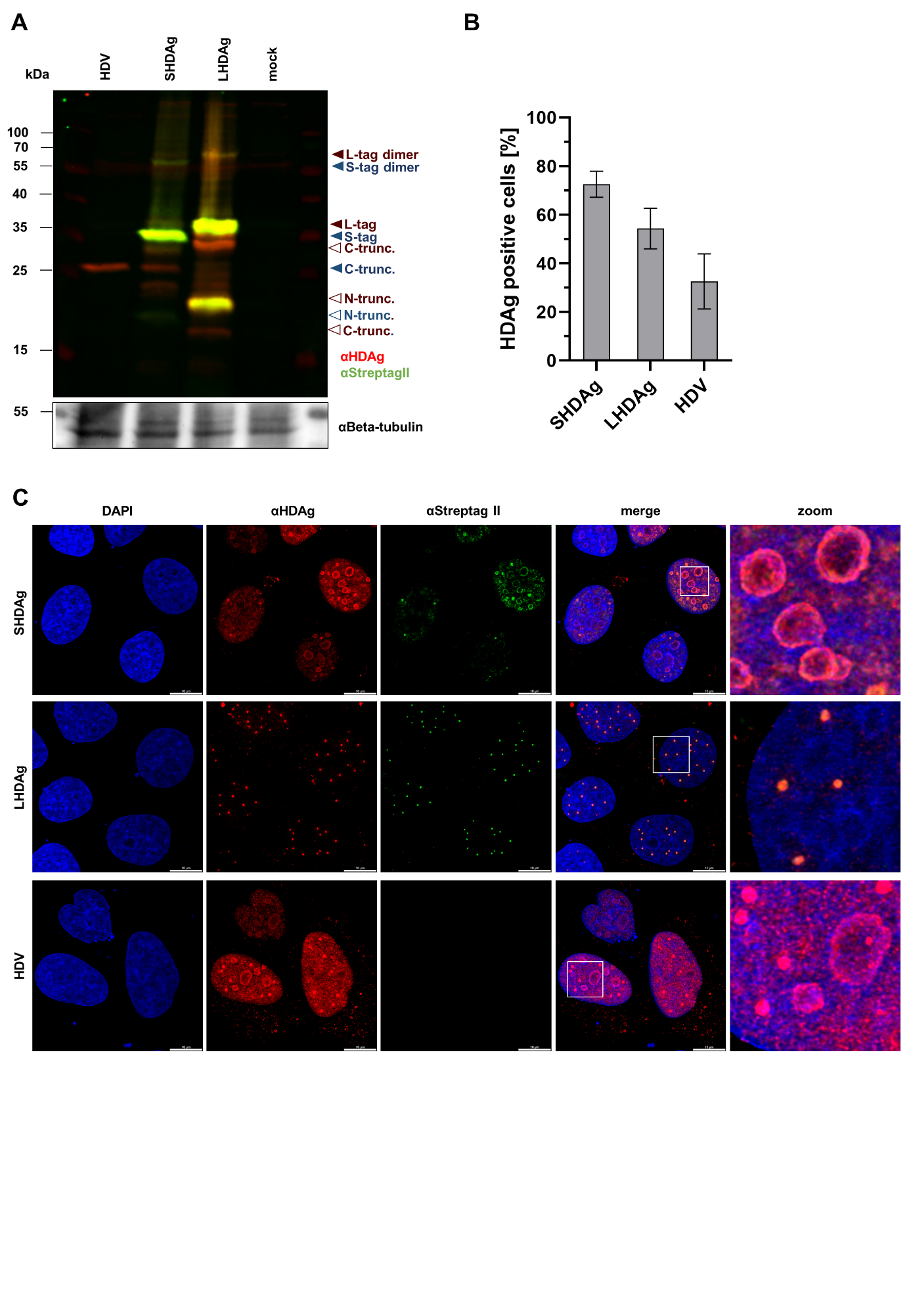
**Figure S1: Selective overexpression of Streptag II tagged S and LHDAg results in differentiated protein fragmentation and subnuclear localization. (A)** Representative WB of cell lysates of Streptag II tagged S/L-HDAg-expressing and HDV-replicating Huh7 cells at 3 dpt; Western blot was probed simultaneously with rabbit derived anti-HDAg specific polyclonal antibody (red) and mouse derived anti-Streptag II specific monoclonal antibody (green). Signals were detected with two different fluorophore conjugated antibodies specific to respective primary antibodies. Displayed is the overlay of both antibody signals. The arrows represent different isoforms. Truncated versions of respective isoforms are indicated in the same color as the full-length protein; L-tag: full-length, tagged LHDAg (monomeric or dimeric); S-tag: full-length, tagged SHDAg (monomeric or dimeric); S: endogenous SHDAg in HDV replicating cells; C-trunc.: C-terminally truncated; N-trunc.: N-terminally truncated. **(B)** Transfection efficiency of selective S/L HDAg expression and expression by HDV replication. Transfection efficiencies were determined by immunofluorescence analysis. HDAg-positive cells were detected using a polyclonal anti-HDAg antibody and total cells detected by DAPI staining. Displayed relative HDAg-positive cell count was obtained by normalizing the absolute HDAg-positive cell count to the total cell count in three independent fields. **(C)** Representative immunofluorescent stains of Huh7 cells expressing Streptag II tagged L-/SHDAg or HDV-replication at 3 dpt; Transfected cells were costained with an anti-HDAg antibody and an anti-Streptag II antibody. blue: DAPI; red: HDAg; green: Streptag II. Scalebar: 10 µm
